# Aetiology of pneumonia in the ICU: the need for early Gram-negative cover

**DOI:** 10.1186/cc9641

**Published:** 2011-03-11

**Authors:** A Khanna, H Al-shather, M Chawla, R Gibbs

**Affiliations:** 1 Musgrove Park Hospital, Somerset, UK; 2 Nottingham City Hospital, Nottingham, UK

## Introduction

Pneumonia remains one of the commonest infectious causes of intensive care unit (ITU) admissions. Despite recent advances, mortality in the ITU from this diagnosis remains around 50% [1]. Early targeted antibiotic therapy to minimise the development of ventilator-associated pneumonia is recommended [2]. This requires an updated knowledge of aetiology of this common diagnosis in ITU settings.

## Methods

We conducted a retrospective cohort study into 200 consecutive admissions to our ITU with coded diagnosis of pneumonia. Baseline patient characteristics microbiological diagnosis, disease severity and mortality outcomes were studied.

## Results

The average patient age in this cohort was 58 years (range 11 to 90 years). The male to female ratio was 1.35:1. All of the patients were admitted to ITU within 48 hours of their hospital admission, mainly due to worsening respiratory failure. Out of the total of 200 cases, microbiological isolates were identified in 110 (55%). Eighty-five isolates were deemed likely to be pathogenic (42.5%) while 25 (12.5%) were likely to be the result of antibiotic use (candida and coliforms species in sputum). Gram-negative bacteria were responsible for 50.9% isolates. *Streptococcus pneumoniae *remained the single most common isolate (28/110; 25.4%). *Pseudomonas *species (23/110; 20.9%) and *Haemophilus influenzae *(11/110; 10%) were the second and third most common isolates. *Pseudomonas *infection was more often associated with advanced age and existing lung pathology. *Staphylococcus aureus *was isolated in 8.1% (9/110) with one confirmed as methicillin resistant (MRSA). Atypical organisms (Legionella 2.7%, mycoplasma spp. 0.9%) and fastidious organisms (*Stenotrophomonas maltophilia *2.7%) were also isolated. Other organisms isolated included enterobacter cloacae, *Citrobacter koseri*, *Streptococcus *Group A, *Haemophilus parainfluenzae*, *Moraxella catarrhalis *and *Kleibsella *species. Mortality amongst our patients was 28.5% (57/200). This was comparable with previously published findings.

## Conclusions

Whilst the aetiology of pneumonia in our cohort is similar to that previously reported [3], the incidence of Gram-negative organisms is much higher. This, if reconfirmed, may have important implications in designing targeted antibiotic therapy for pneumonia in ITU settings.
